# Variation in the Concentration of *Tilia* spp. Pollen in the Aeroplankton of Lublin and Szczecin, Poland

**DOI:** 10.3390/plants12061415

**Published:** 2023-03-22

**Authors:** Elżbieta Weryszko-Chmielewska, Krystyna Piotrowska-Weryszko, Tomasz Wolski, Aneta Sulborska-Różycka, Agata Konarska

**Affiliations:** 1 Department of Botany and Plant Physiology, University of Life Sciences, Akademicka 15, 20-950 Lublin, Poland; 2 Institute of Marine & Environmental Sciences, University of Szczecin, Felczaka 3c, 71-412 Szczecin, Poland

**Keywords:** lime, pollen seasons, annual pollen sum, global warming

## Abstract

Although lime trees have numerous benefits, they can pose a threat to allergy sufferers during the flowering period, as their pollen exhibits allergenic properties. This paper presents the results of 3 years of aerobiological research (2020–2022) carried out with the volumetric method in Lublin and Szczecin. A comparison of the pollen seasons in both cities revealed substantially higher concentrations of lime pollen in the air of Lublin than of Szczecin. In the individual years of the study, the maximum pollen concentrations were approximately 3-fold higher, and the annual pollen sum was about 2–3 times higher in Lublin than in Szczecin. Considerably higher lime pollen concentrations were recorded in both cities in 2020 than in the other years, which was probably associated with the 1.7–2.5 °C increase in the average temperature in April compared to the other two years. The maximum lime pollen concentrations were recorded during the last ten days of June or at the beginning of July in both Lublin and Szczecin. This period was associated with the greatest risk of pollen allergy development in sensitive subjects. The increased production of lime pollen in 2020 and in 2018–2019 with the increase in the mean temperature in April, reported in our previous study, may indicate a response of lime trees to the global warming phenomenon. Cumulative temperatures calculated for *Tilia* may serve as a basis for forecasting the beginning of the pollen season.

## 1. Introduction

Lime (*Tilia* spp.) trees are apicultural, medicinal, cosmetic, and ornamental plants. Due to the abundant nectar production and numerous bee visits to their flowers, they are regarded as one of the best melliferous plants [1,2]. Insects collect both nectar and pollen from lime flowers. Honeybee visitors form compact, light yellow or intense yellow pollen baskets [3]. Lime flowers and leaves are used in medicine [4,5], whereas the flowers, leaves, and fruits are applied in cosmetology [6,7,8].

Lime species growing in natural sites in Poland are represented by *Tilia cordata* Mill., *Tilia platyphyllos* L., and the less common *Tilia* × *europea* L., which is a hybrid of the other two species. Additionally, several other species are cultivated, with the most frequent being *Tilia tomentosa* Moench and *Tilia × euchlora* K. Kohl [9]. *Tilia cordata* can live up to 1000 years [10]. It produces its first flowers at between 20 and 30 years of age [3].

Lime flowers are pentamerous and consist of sepals with a nectary at the base, a greenish or yellow corolla, numerous stamens, and one fivefold pistil. The androecium consists of whorls of five stamens. On average, there are 30 and 53 stamens in *T. cordata* and *T. platyphyllos* flowers, respectively [11]. Lime flowers are protandrous, usually with a 1–2 day stamen phase and a 4–8 day pistil phase. One *T. cordata* flower produces 43,000 pollen grains, whereas 200,000 grains are produced by 1 inflorescence [3]. *Tilia platyphyllos* flowers earlier, i.e., during the first ten days of June or during a ten-day period in the middle of June, while *T. cordata* starts blooming about two weeks later [11].

Some pollen produced by lime stamens persists in the air. Pollen grains of various *Tilia* species are classified as medium size (29.1–49.4 µm) [12]. Their greatest concentrations are recorded near pollen-producing trees [13]. In sensitive subjects, they trigger symptoms of pollen allergy. Pollen grains of *T. cordata* causes allergic rhinitis, asthma, rhinoconjunctivitis, cough, and allergic contact dermatitis [14,15]. According to the classification of the Polish Society of Allergology, *Tilia* pollen is classified as class 2—moderate allergenicity, similar to *Fraxinus*, *Populus*, and *Quercus* pollen. Various researchers have also determined the allergenicity of *Tilia* pollen as moderate [16,17,18]. Cases of allergy to lime pollen were described as early as 1941 [19]. A detailed description of a case of severe symptoms of *Tilia* pollen allergy in Spain was published by Mur et al. [14]. Similarly, the authors of the present study can confirm that sensitive subjects who stay in the vicinity of *Tilia* trees may develop strong symptoms of pollen allergy (unpublished information). In Poland, there are no data of subjects that are allergenic to the pollen of these taxa. At the same time, in Portugal, pollen of linden was found to be one of the most representative aeroallergens, resulting in sensitization in 11.4 % of 557 pediatric patients [20]. Moreover, in Turkey, sensitization to *Tilia* was found in 5.5% of children and in 42.5% of allergic adults [21,22]. Unfortunately, no allergens from *Tilia* pollen have been characterized to date [23].

In recent years, an increasing frequency of phenomena associated with climate change that have a negative impact on nature and humans can be observed. These threats are caused by extreme meteorological phenomena, e.g., several-day-long rainfall, heat waves, and droughts [24,25,26,27]. One of these problems is the high variability of the characteristics of plant pollen seasons [28,29,30]. The beginning and length of the pollen season as well as the annual sums of lime pollen grains were reported to vary substantially in Lublin in 2001–2018. An impact of global warming on the onset of the lime pollen season has been demonstrated. Acceleration of the onset of lime flowering and pollen release by 2 weeks and a significant increase in the airborne pollen concentration of this taxon were shown by data from 2018 [11]. Lime pollen concentrations and the course of pollen seasons were also shown to differ considerably between Polish cities [31].

The aim of the study was to compare the course of lime pollen seasons in Lublin and Szczecin in 2020–2022, taking into account the main parameters of the season: the start and end date, duration, maximum concentrations, and annual pollen sum. The dynamics of the pollen seasons in both cities and the impact of weather conditions on airborne pollen concentrations were analyzed as well. Additionally, the present results were compared with data from Lublin obtained in previous years (2001–2019) [11,31].

## 2. Results

### 2.1. Characteristics of Lime Flowers

**Figure 1:**
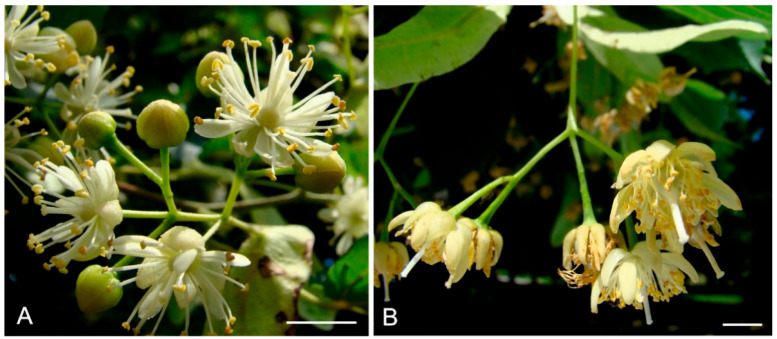
Inflorescences of two *Tilia* species: (**A**) *Tilia cordata*; (**B**) *Tilia platyphyllos*. Scale bars = 0.5 cm (**A**,**B**).

The structure of lime flowers exhibits traits typical of both insect-pollinated and wind-pollinated plants. The presence of the perianth, hermaphroditism, intense fragrance, and nectar production are the typical features of insect-pollinated flowers. Traits indicating simultaneous anemophily include the presence of drooping inflorescences in many *Tilia* species, weak coloration of the perianth, well-exposed stamens, and pistils longer than the corolla petals (Figure 1A,B).

**Figure 2:**
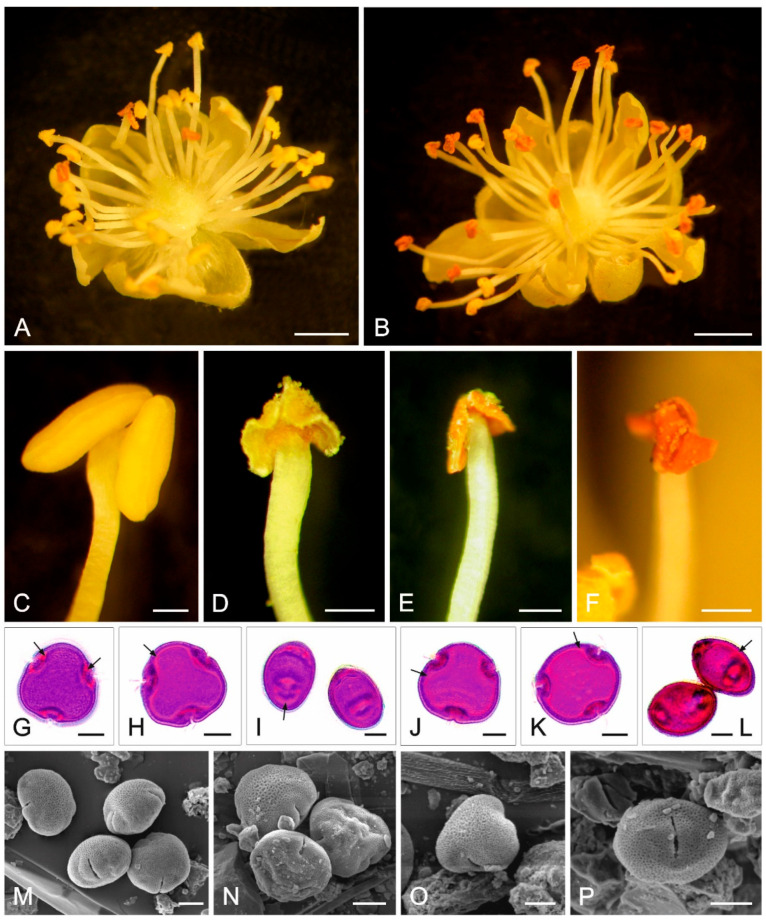
Lime flowers, stamens, and pollen grains. (**A**) *T. cordata* flowers at the start and (**B**) end of pollen release; (**C**–**F**) *T. cordata* stamens: (**C**) before pollen release, (**D**,**E**) during pollen release, (**F**) after pollen release; (**G**–**I**) *T. cordata* pollen grains stained with basic fuchsine (light microscope, LM); (**J**–**L**) *T. platyphyllos* pollen grains stained with basic fuchsin (LM). (**G**–**L**) Arrows show intine stained red; (**M**–**P**) pollen grains of the different *Tilia* species contaminated by biological material and dust particles from aerobiological preparations (scanning electron microscope, SEM). Scale bars = 2.5 cm (**A**,**B**); 1 mm (**C**–**F**); 10 µm (**G**–**P**).

The numerous stamens growing in several whorls in the plate-shaped *T. cordata* flowers mature before the pistils (protandry). Their maturation proceeds non-simultaneously in the flower, which is evidenced by the different colors of the anthers (Figure 2A). After pollen release, the anthers change color from yellow to orange (Figure 2B). Stamens from different stages of pollen release and a visible color change are shown in Figure 2C–F. The pollen grains of *T. cordata* (Figure 2G–I) and *T. platyphyllos* (Figure 2J–L) are tricolporate with short furrows. The exine forms a reticular pattern on the surface, on which contaminants of various origins were visible in aerobiological preparations with the scanning electron microscope (Figure 2M–P). The three characteristic pores and furrows are located in the equatorial plane of the pollen grain (Figure 2G–L). The treatment of lime pollen grains with basic fuchsin yielded differentiated coloration of the sexine and nexine layers with visualization of very small cavities in the exine. In addition, the staining facilitated easy localization of the intine, i.e., a red-stained pectinaceous layer (Figure 2G–L).

### 2.2. Characteristics of Pollen Seasons

In 2020–2022, the lime pollen seasons in Lublin started between 17 June and 21 June and lasted on average 61 days (Table 1). The pollen seasons of this taxon in Szczecin in the same years started slightly later, i.e., between 20 June and 23 June. The pollen seasons in this city were shorter than in Lublin, as they lasted on average 48 days. In both cities, we recorded the presence of Poaceae, *Urtica*, *Rumex*, *Plantago*, and Chenopodiaceae/Amaranthaceae pollen grains in the analyzed periods.

**Figure 3:**
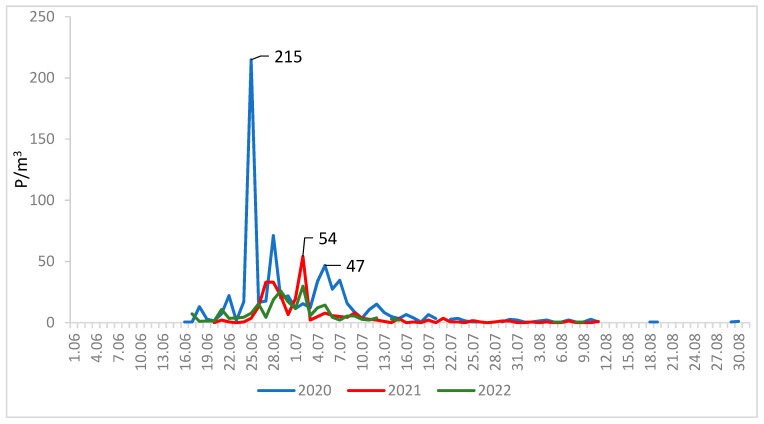
Daily concentrations of *Tilia* pollen in the air of Lublin in 2020–2022.

**Figure 4:**
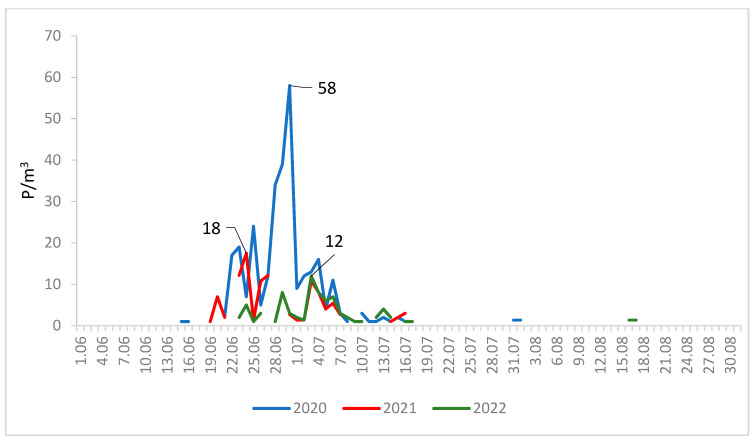
Daily concentrations of *Tilia* pollen in the air of Szczecin in 2020–2022.

The maximum concentrations of lime pollen grains were much higher in Lublin (on average 100 P/m^3^) than in Szczecin (on average 29 P/m^3^) (Figure 3 and Figure 4). In 2020, the maximum pollen concentrations in both cities substantially exceeded the values obtained in the other two years. The maximum concentration of pollen grains in 2020 was 215 P/m^3^ (25 June) in Lublin and 58 P/m^3^ (30 June) in Szczecin (Table 1).

**Figure 5:**
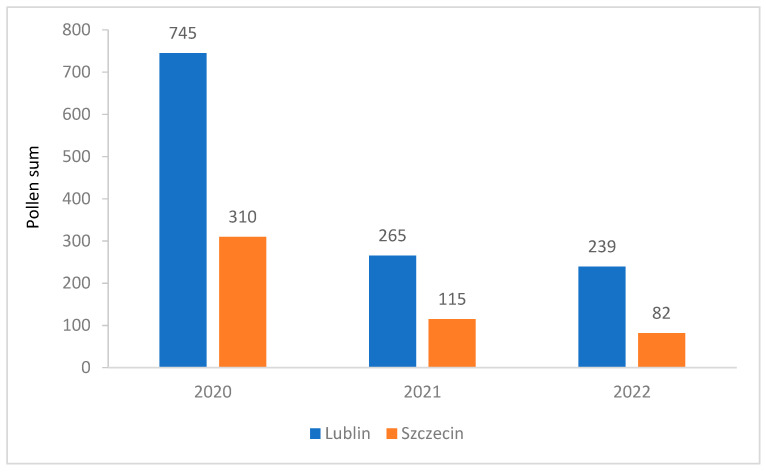
Comparison of the annual sum of *Tilia* pollen concentrations in Lublin and Szczecin in the years 2020–2022. Annual pollen sum of *Tilia* pollen in Lublin and Szczecin in 2020–2022.

Similarly, the annual pollen sums were over twofold higher in Lublin (on average 416) than in Szczecin (on average 169). The highest annual pollen sums were recorded in 2020 in both Lublin (745) and Szczecin (310) (Table 1, Figure 5).

**Figure 6:**
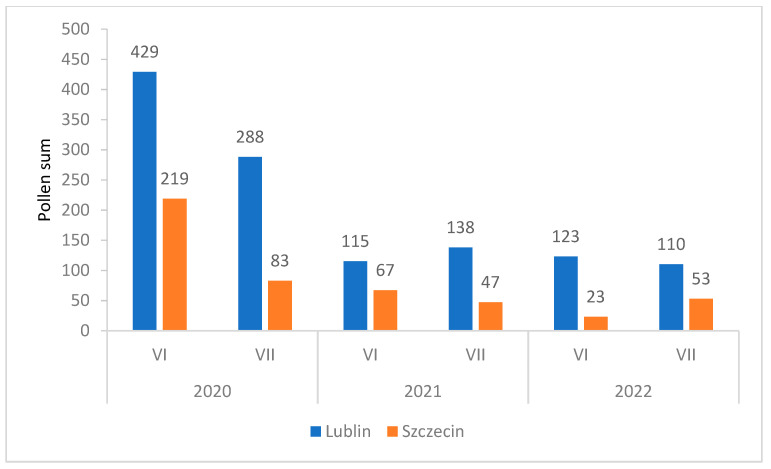
Comparison of the sum of *Tilia* pollen concentrations in Lublin and Szczecin in the years 2020–2022. Monthly pollen sum of *Tilia* pollen in Lublin and Szczecin in 2020–2022.

In the study years, lime pollen was recorded in the air in June and July. In both cities, a greater abundance of the pollen of this taxon was detected in June (Figure 6).

### 2.3. Comparison of the Dynamics of Pollen Seasons in Lublin and Szczecin

The curves showing the course of the lime pollen seasons are characterized by the presence of many peaks (Figure 3 and Figure 4), which are associated with the different terms of flowering of several *Tilia* species present in the analyzed area.

**Figure 7:**
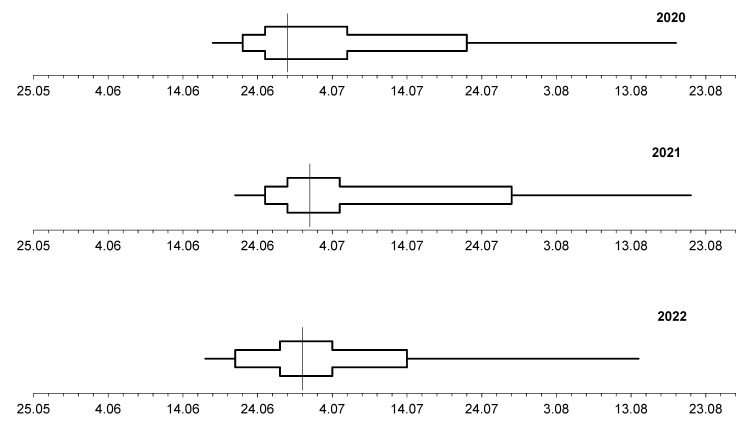
Dynamics of the *Tilia* pollen seasons in Lublin, 2020–2022.

**Figure 8:**
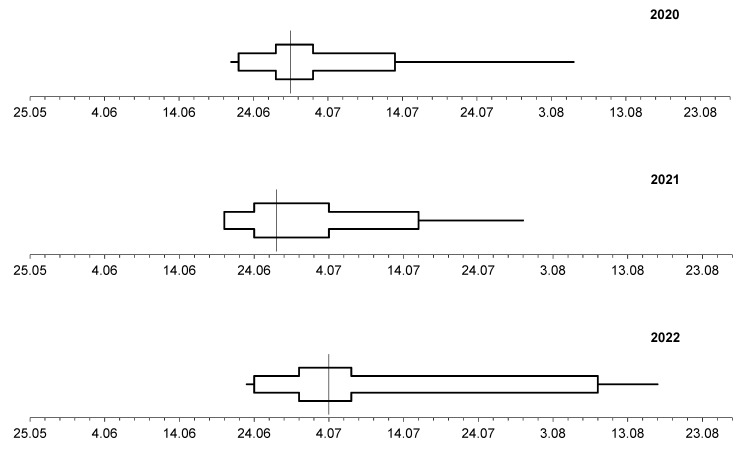
Dynamics of the *Tilia* pollen seasons in Szczecin, 2020–2022.

The preliminary comparison of the dynamics of pollen seasons in Lublin and Szczecin during the three study years showed considerable differences between the seasons in the individual years and in the different cities (Figure 7 and Figure 8).

In Lublin and Szczecin, 50% of the annual airborne *Tilia* pollen sum was reached during the last 10 days of June. An exception was noted in Szczecin in 2022, where this value of the parameter was recorded during the first 10 days of July. During the study years, the stages with cumulative pollen grain percentages ranging from 25% to 75% had different lengths in both cities (Figure 7 and Figure 8). This indicated variations in the length of the phase of release of large amounts of lime pollen. This phase had the longest duration in Lublin in 2021 and in Szczecin in 2022.

The comparison of the dynamics of the lime pollen seasons indicated that they were asymmetric. Since the stages after reaching the cumulative percentage of 50% pollen grains were longer that the preceding ones, the pollen seasons were classified as right-skewed.

### 2.4. Impact of Weather Factors on Lime Pollen Concentrations

Since thermal differences may have contributed to the increased concentrations of lime pollen in 2020, we analyzed the average temperatures in April and found that they were higher by 2.3–2.4 °C and 1.7–2.5 °C in both Lublin (8.4 °C) and Szczecin (8.9 °C) in 2020, respectively, than in the other two years (Table 2).

**Figure 9:**
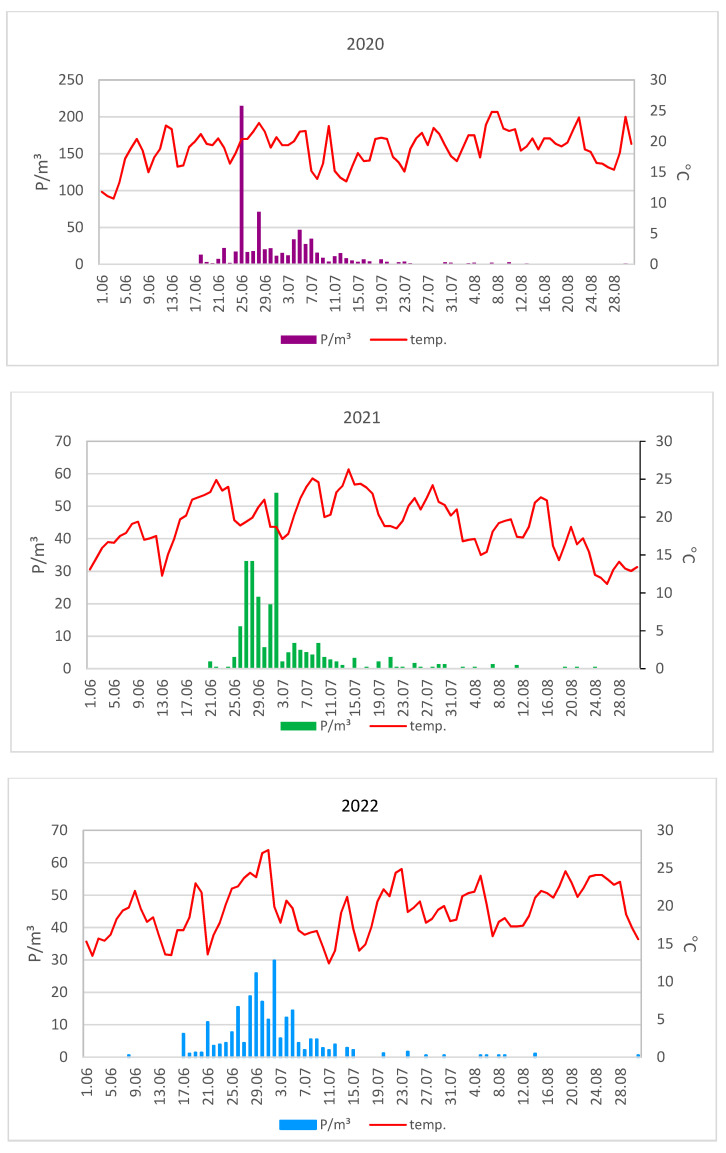
Daily concentrations of *Tilia* pollen in the air of Lublin in 2020–2022 versus mean daily air temperature.

**Figure 10:**
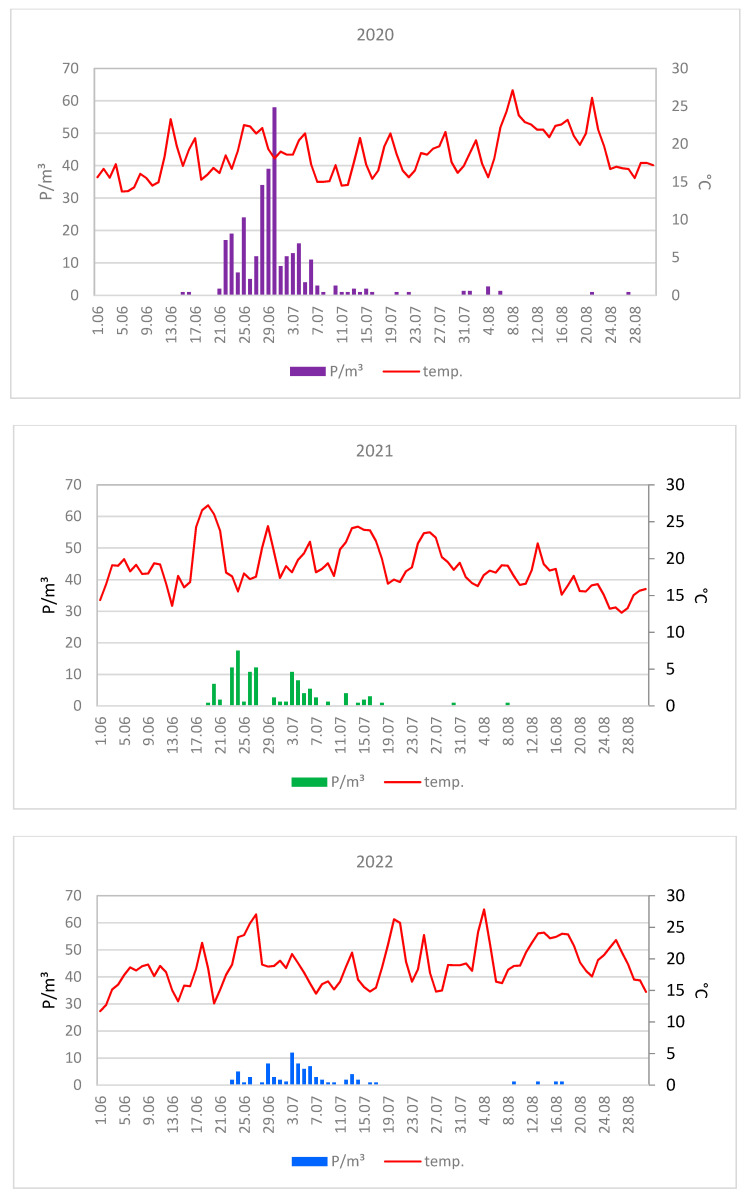
Daily concentrations of *Tilia* pollen in the air of Szczecin in 2020–2022 versus mean daily air temperature.

An analysis of the graphs of temperature and pollen concentrations during the lime pollen seasons showed that the occurrence of maximum pollen concentrations was preceded by a several-day period of an average daily temperature of 20–27 °C in Lublin and Szczecin in the individual years (Figure 9 and Figure 10).

As indicated by the statistical analysis, no significant effect of the temperature, rainfall, and relative air humidity on the presence of lime pollen grains in the air was found during the pollen season (Table S1).

### 2.5. Cumulative Temperature

In comparison with Szczecin, Lublin was characterized by more similar values of cumulative temperatures at the air temperatures of >0 °C calculated in the study years from both 1 January and 1 March (Table 3). This fact is confirmed by the lower coefficients of variation shown in the table. The average values of cumulative temperatures in Szczecin were higher than in Lublin, i.e., the average cumulative temperature since 1 January was 1351.8 °C in Szczecin and 1082.4 °C in Lublin. In the cumulative series created for the temperatures of >5.5 °C, more similar values were obtained for Lublin and higher values of the calculated temperature were recorded in Szczecin (Table 4).

## 3. Discussion

The concentrations of lime pollen during the 2020–2022 pollen seasons were substantially higher in Lublin than in Szczecin. This may be related to the close proximity of the measurement sites in Lublin to two parks where lime trees are grown. Additionally, there are many *T. cordata* trees surrounding the measurement site and in the two neighboring streets. As reported in a previous study, lime trees are dominant species in Lublin parks [32]. There were only a few trees of this taxon in close proximity to the measuring site in Szczecin. However, a study conducted in Ukraine showed that, due to the lime entomophily, the large number of trees was not reflected in the pollen spectrum in various cities, as the amount of lime pollen accounted for less than 1% [33,34].

In 2020, the proportion of lime pollen in the total annual sum of pollen in the aeroplankton was 0.86% in Lublin and 0.47% in Szczecin. A similar content of *Tilia* pollen, i.e., on average 0.8%, was recorded during several years in the air of Salamanca (Spain) [35]. Studies conducted in Russia showed lime pollen originating from long-distance transport, which was detected at a distance of 80–150 km [36].

In Rzeszów, pollen concentrations of various trees (e.g., lime trees) growing in urban parks were compared with the concentration of pollen of this taxon on the roof of a building. It was shown that the lime pollen concentration in the parks measured at a low height was higher than that measured on the roof of the 12 m high building [13].

Many researchers regard the allergenicity of *Tilia* pollen as moderate [16,17,18,37]. In contrast, Cariñanos and Casares-Porcell [38] argue that the allergenic index of this taxon may be high, as *Tilia* species are frequently planted in parks and alley greenery in the temperate climate of Europe. Furthermore, the surface of *Tilia* pollen grains observed by us were contaminated by biological material and suspended dust particles, which have an adjuvant effect, enhancing the organism immune response and intensifying the symptoms of pollen allergy [39,40].

Studies conducted in Turkey reported a significantly high content of allergenic proteins in *T. cordata* pollen [41]. The present study indicates that the greatest risk of development of pollen allergy triggered by lime pollen may be encountered during the third ten days of June and at the beginning of July.

*Tilia* species are regarded as thermophilic taxa [42]. As shown by the literature data, woody plants are highly responsive to environmental changes [43]. Flowering phenology is largely influenced by temperature [44,45]. The present study demonstrated that the cumulative temperature had an impact on the onset of *Tilia* pollen season in both Lublin and Szczecin, with more similar values in the years of the study recorded in Lublin. We found that the cumulative temperature for *Tilia* calculated from January 1 to the beginning of the pollen season at temperatures of >0 °C was on average 1082.4 °C in Lublin and 1351.8 °C in Szczecin. These differences may be associated with the different microclimate conditions in these cities. In studies of 14 woody taxa, it was shown that cumulative temperature was an essential factor for the start and the end of flowering [46]. Many researchers highlight the possibility of using cumulative temperature to forecast the beginning of pollen seasons [47,48,49] and different phenological phases in woody plants [50].

Since lime flower buds are formed in early spring in the year of flowering, the flower development rate depends on the temperature in the spring months [51,52]. The present study showed that the average temperature in April in Lublin and Szczecin was 1.7–2.5 °C higher in 2020 that in 2021 and 2022. The higher temperatures in April 2020 were accompanied by higher concentrations of lime pollen in the air than in the other two years. Therefore, it can be assumed that the temperature increase in April 2020 may have influenced the production of *Tilia* pollen in both cities. The annual pollen sum in Lublin was 740 in 2020 but only 261 and 239 in 2021 and 2022, respectively. Similar findings were reported in Lublin in 2018, i.e., the higher temperatures in April and May than in the previous years contributed to substantially higher amounts of airborne lime pollen, with an annual pollen sum of 968 and an acceleration in flowering and pollen release by 14 days. The annual pollen sum in 2001–2018 had an average value of 332 [11]. However, a high annual pollen sum of 844 was recorded in 2019, which was characterized by higher temperatures in April [31].

In 2018–2020, a high peak value of *Tilia* pollen was also determined in Lublin (243, 150, and 215 P/m^3^, respectively), while the mean value of this characteristic in the 2001–2018 pollen season was 54.2 P/m^3^. The peak value recorded in Lublin in 2020 on 25 June was similar to the mean value of this parameter in this city (26 June) in 2001–2018. Earlier dates of the maximum concentration of lime pollen were noted in Lublin in 2018 and 2019, i.e., 9 June and 16 June, respectively [11,22].

Similar to some other tree species, *Tilia* has responded to global warming and the related instability of average air temperatures from year to year. Phenological observations carried out by other authors revealed an 11-day acceleration in the bud burst phase in lime trees, which was associated with the warmer weather prevailing in recent years [53,54].

This finding confirms the results of our previous aerobiological studies conducted in 2001–2018, where accelerated lime pollen release and an increase in the concentration of pollen grains were reported [11]. The present study also confirmed the response of lime trees to the increase in temperature in April 2020, when the recorded pollen amounts in the two studied cities were clearly higher in comparison with the values noted in 2021 and 2022.

## 4. Materials and Methods

### 4.1. Vegetation of Trees in the Surroundings of the Pollen Samplers

In Lublin, over a dozen *Tilia* trees are growing around the university building, where the measurement site was located. Many trees of this taxon were planted along streets located in close proximity to the pollen samplers. Additionally, lime trees are dominant plants in two city parks located nearby [32]. There are also representatives of *Quercus*, *Acer*, *Aesculus*, *Betula*, *Populus*, *Sorbus*, *Fraxinus*, *Fagus*, *Salix*, *Gleditschia*, *Pinus*, *Picea*, *Taxus*, *Thuja*, and *Juniperus* growing here. In Szczecin, there are several lime trees in the vicinity of the building with the measurement site. *Populus*, *Betula*, and *Morus* plants grow in the neighboring street. At a distance of 0.5 km, there are numerous *Platanus acerifolia* plants. There is also a nearby park dominated by representatives of the genera *Acer* as well as *Quercus*, *Fagus*, *Populus*, *Fraxinus*, *Tilia*, *Carpinus*, *Salix*, *Betula*, *Abies*, *Picea*, *Pinus*, and *Taxus*.

### 4.2. Plant Material

Two *Tilia* species growing in the Botanical Garden of Maria Curie-Skłodowska University in Lublin (51°15′44′′ N, 22°30′48′′ E) were used for morphological observations: *Tilia cordata* Mill. and *Tilia platyphyllos* L. Photographic documentation was made using a Sony α 6000 camera. Inflorescences (n = 10) of the analyzed species were collected randomly.

### 4.3. Microscopy Observations

Flower morphology was observed under an STM 800 stereomicroscope (mikroLAB). Photographs were taken using a Nikon Coolpix 4500 camera (Nikon, Tokyo, Japan).

Fresh pollen from anthers of both species was collected and glycerin–gelatin preparations with the addition of basic fuchsin were made [55]. Basic fuchsin stains the exine and the intine. Photographic documentation was made with the use of a Nikon Eclipse 400 light microscope (Nikon, Tokyo, Japan) coupled to an Olympus DP23-CU camera (Olympus, Tokyo, Japan) working with CS-EN-V3 cellSens Entry V3 software.

*Tilia* pollen grains were also observed using a scanning electron microscope (SEM). Aerobiological samples were collected in 2020 using Durham devices located at a height of 2 m in the Botanical Garden of Maria Curie-Skłodowska University in Lublin. Slides covered with double-sided adhesive tape were placed in the devices. Fragments of the tape with biological aerosol samples were coated with gold using an Emitech SC 7640 sputter coater (Polaron, Newhaven, East Sussex, UK) without application of standard preparatory procedures used in scanning electron microscopy. The preparations were observed and photographed in a TESCAN/VEGA LMU (Tescan, Brno, Czech Republic) scanning electron microscope equipped with a TESCAN attachment for digital processing of microscopic images.

### 4.4. Aerobiological Study

**Figure 11:**
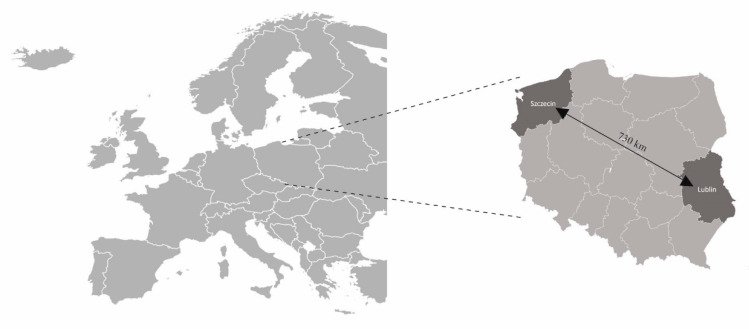
The location of the study areas in two cities in Poland.

The lime pollen concentration in the air was measured in Lublin and Szczecin (Poland) in 2020–2022 (Figure 11). The temperate climate of the Lublin region exhibits a clear influence of continental air masses. The mean annual air temperature in Lublin in 1961–2020 reached 7.8 °C, and the mean annual precipitation sum was 591 mm [56]. Western Pomerania, where Szczecin is located, is characterized by a temperate climate with prevailing western, northwestern, and northern winds. The multitude of water reservoirs and the large forest area contribute to the high air humidity in this region. The mean annual air temperature in Szczecin in 1961–2020 was reported to reach 9.0 °C, and the mean annual precipitation sum was 547 mm [56].

Hirst-type devices (Lanzoni VPPS 2000) were used to collect pollen samples from the air. The measurement site in Lublin was located close to the city center (51°14′37′′ N and 22°32′5′′ E; 197 m a.s.l.) on a flat roof of the building of the University of Life Sciences at a height of 18 m above the ground. In Szczecin, the sampler was placed on the roof of the building of the Institute of Biology at a height of 21 m above ground level in Śródmieście district (53°26′22′′ N and 14°32′52′′ E; 52 m a.s.l.).

The results were expressed as the number of pollen grains in 1 m^3^ of air per day (P/m^3^) [57]. The 98% method was used to determine the pollen season dates [58]. The start of the season was defined as the date when 1% of the seasonal cumulative pollen concentration was trapped, and the date with the cumulative pollen concentration of 99% was considered the end of the season.

**Figure 12:**
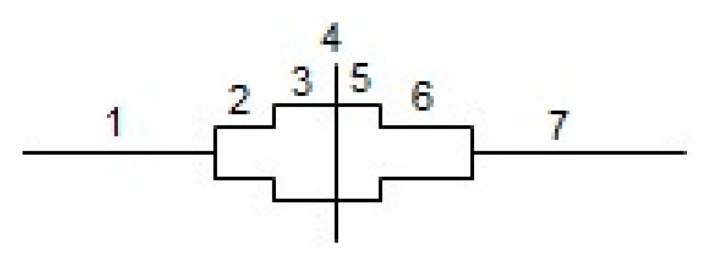
Graphical representation of the dynamics of the achievement of the annual pollen sum. Stages of the pollen season: (1) 1–5%, (2) 5–25%, (3) 25–50%, (4) 50%, (5) 50–75%, (6) 75–95%, (7) 95–99%.

The following parameters of the pollen season were analyzed: start, end, and duration; maximum pollen concentration (peak value); date of the maximum concentration; and annual pollen sum. The dynamics of reaching the annual sum of lime pollen grains was studied by determination of the duration of the successive pollen season stages corresponding to the achievement of a specified percentage share of the annual pollen sum [59] (Figure 12).

The relationships between the course of the season and selected meteorological factors (mean temperature, humidity, and rainfall) were determined using Spearman’s correlation analysis. Data on average daily values of air temperature, relative humidity, and precipitation were obtained from the Institute of Meteorology and Water Management. The statutory task of this institute is to conduct meteorological measurements throughout the country.

Since the development and maturation of anthers depend on the temperature, and they open after absorbing a certain dose of thermal energy [60,61], we calculated cumulative (effective) temperatures for *Tilia* growing in Lublin and Szczecin. These temperatures are used to forecast the beginning of pollen seasons [48]. To this end, the temperatures of >0 °C and >5.5 °C were summed up from January 1 and March 1 each year to the day of the onset of the pollen season, and, after calculation of the coefficients of variation, the most similar values of cumulative temperatures in the individual years were selected for *Tilia*.

## 5. Conclusions

The substantially higher *Tilia* pollen concentrations recorded in Lublin than in Szczecin during the study are probably a result of the differences in the abundance of lime trees in the vegetation surrounding the measurement sites in the analyzed cities.

The values of cumulative temperature for *Tilia* recorded in Lublin in the years of the study exhibit slight variation and can be used to forecast the beginning of the pollen season.

The considerably higher amounts of lime pollen revealed by the pollen monitoring in 2020 than in the other two years in both cities are possibly related to the impact of the higher temperatures prevailing in the spring (April) on plants, i.e., during the intensive development of flowers in this taxon. Since the information on the increased *Tilia* pollen production and the temperature rise in April applies only to the three research years (2018, 2019, 2020), further aerosol analyses are required to confirm this phenomenon.

## Figures and Tables

**Table 1:** Characteristics of linden pollen seasons in the years 2020–2022.

Pollen Season Parameters	Lublin			Szczecin		
	2020	2021	2022	2020	2021	2022
Pollen season period by the 98% method	18.06–19.08	21.06–21.08	17.06–14.08	21.06–6.08	20.06–30.07	23.06–17.08
Season duration	63	62	59	47	41	56
Peak value (P/m^3^)	215	54	30	58	18	12
Peak date	25.06	2.07	2.07	30.06	24.06	3.07
Annual pollen sum	740	261	239	310	115	82
Number of days with concentration ≥ 5 P/m^3^	25	12	14	14	8	6

**Table 2:** Average air temperature in April in individual years, 2020–2022.

	Lublin			Szczecin		
	2020	2021	2022	2020	2021	2022
Average air temperature in April	8.4	6.0	6.1	8.9	6.4	7.2

**Table 3:** Cumulative temperature >0 °C for Lublin and Szczecin prior to the beginning of the *Tilia* pollen season in 2020–2022.

Year	Temperature from 1 January to the Beginning of the Pollen Season (°C)		Temperature from 1 March to the Beginning of the Pollen Season (°C)	
	Lublin	Szczecin	Lublin	Szczecin
2020	1129.6	1454.5	1013.2	1145
2021	1057.3	1188.8	1000.8	1074.3
2022	1060.3	1412.2	958	1175.5
Average	1082.4	1351.8	990.7	1131.6
SD	40.9	142.8	29.0	51.9
V%	3.8	10.6	2.9	4.6
Range	72.3	265.7	55.2	101.2

**Table 4:** Cumulative temperature >5.5 °C for Lublin and Szczecin prior to the beginning of the *Tilia* pollen season in 2020–2022.

Year	Temperature from 1 January to the Beginning of the Pollen Season (°C)		Temperature from 1 March to the Beginning of the Pollen Season (°C)	
	Lublin	Szczecin	Lublin	Szczecin
2020	970	1244.6	942.4	1072.7
2021	927.3	1029.5	908.5	957.9
2022	907.6	1224.6	887.6	1103.7
Average	935.0	1166.2	912.8	1044.8
SD	31.9	118.8	27.7	76.8
V%	3.4	10.2	3.0	7.4
Range	62.4	215.1	54.8	145.8

## Data Availability

All relevant data is found in this article.

## Supplementary Materials

The following supporting information can be downloaded at: https://www.mdpi.com/article/10.3390/plants12061415/s1, Table S1: The list of Spearman’s correlations between the daily pollen count and meteorological factors in Lublin and Szczecin (2020–2022).
