# Supplementary material for: Variation in the Concentration of Tilia spp. Pollen in the Aeroplankton of Lublin and Szczecin, Poland
Source: Plants (Basel). 2023 Mar 22;12(6):1415. doi: 10.3390/plants12061415 (PMC10053843; doi:10.3390/plants12061415)
Supplement: Supplementary file 1 [file plants-12-01415-s001.zip › plants-2236949-supplementary.pdf]

Table S1. The list of Spearman's correlations between the daily pollen count and meteorological factors in Lublin and Szczecin (2020-2022).

Meteorological parameters	Spearman coefficient	
	Lublin	Szczecin
Mean temperature	0,00778	-0,04277
Humidity	0,15684*	0,16791*
Rainfall	0,13390	0,21689**

Level of significance * 0.05, ** 0.01
